# Interpretation of Whole-Genome Sequencing for Enteric Disease Surveillance and Outbreak Investigation

**DOI:** 10.1089/fpd.2019.2650

**Published:** 2019-07-09

**Authors:** John M. Besser, Heather A. Carleton, Eija Trees, Steven G. Stroika, Kelley Hise, Matthew Wise, Peter Gerner-Smidt

**Affiliations:** Division of Foodborne, Waterborne, and Environmental Diseases, Centers for Disease Control and Prevention, National Center for Emerging and Zoonotic Diseases, Atlanta, Georgia.

**Keywords:** molecular epidemiology, foodborne outbreaks, foodborne disease epidemiology

## Abstract

The routine use of whole-genome sequencing (WGS) as part of enteric disease surveillance is substantially enhancing our ability to detect and investigate outbreaks and to monitor disease trends. At the same time, it is revealing as never before the vast complexity of microbial and human interactions that contribute to outbreak ecology. Since WGS analysis is primarily used to characterize and compare microbial genomes with the goal of addressing epidemiological questions, it must be interpreted in an epidemiological context. In this article, we identify common challenges and pitfalls encountered when interpreting sequence data in an enteric disease surveillance and investigation context, and explain how to address them.

## Introduction and Terminology

Detection and investigation of outbreaks through molecular-based surveillance of foodborne pathogens by pulsed-field gel electrophoresis (PFGE) has proven to be a highly cost-effective tool for food safety (Scharff *et al.*, 2016). Whole-genome sequencing (WGS) promises to bring that effectiveness to a new level, and open up new opportunities for understanding trends in antimicrobial resistance, virulence, and foodborne microbial ecology. WGS has made it possible to detect more outbreaks with fewer cases (Jackson *et al.*, 2016; Moura *et al.*, 2017), and to link human illness to specific foods or production environments with greater confidence than ever before. However, early hopes that WGS would drastically simplify the identification of contaminated foods and tracking them to their sources have largely been replaced by the realization that WGS also reveals the vast complexity of microbial interactions with humans, animals, plants, and the environment. In doing so, WGS also opens up the door for answering questions about foodborne pathogens and potential prevention activities that would not have been answerable before.

WGS analysis of foodborne pathogens such as *Salmonella* spp. and *Listeria monocytogenes* has been widely used to identify possible food or environmental sources of outbreaks initially recognized by some other means, for example, laboratory surveillance by less discriminatory methods such as PFGE or by citizen reporting. Increasingly, WGS is being used as a primary outbreak detection tool, allowing detection of widely dispersed outbreaks that might not be otherwise identified (Besser *et al.*, 2018). This is accomplished by comparing the genomes of pathogens isolated from patients to identify clusters of disease that suggest outbreak occurrence, and conducting interviews to find a common exposure, such as consuming the same contaminated food product. WGS and other molecular methods are also used to presumptively link results from routine food monitoring programs, such as those conducted at USDA and FDA through the Genome TrakR network, to seemingly “sporadic” cases (Allard *et al.*, 2016).

WGS also makes it possible to track trends associated with pathogen virulence and antimicrobial resistance, and it is increasingly being explored as a tool for food source attribution (de Knegt *et al*., 2016). The most common analyses used for this purpose are high-quality single-nucleotide polymorphism (hqSNP) analysis (where SNP positions are filtered based on sequence quality, and mobile and phage elements are generally masked), core genome multilocus sequence typing (cgMLST, a gene-by-gene analysis using only core genes), or whole-genome MLST (wgMLST; MLST using both core and accessory genes). Details, relative merits, and limitations of these methods are described elsewhere (Katz *et al.*, 2017; Nadon *et al.*, 2017; Jagadeesan *et al.*, 2019).

Differences between genomes are most commonly visualized as SNP/allele matrices, which provide pairwise comparison information, and phylogenetic trees that predict evolutionary relationships between strains. These data are used to identify disease clusters or clusters between cases, food or environmental isolates. Clusters have classically been defined in terms of space and time (Last, 1983), but WGS has the potential for extending the boundaries of what constitutes a cluster. The word “outbreak” is generally used to describe a cluster when an epidemiological link is identified.

Finally, outbreaks are described as “clonal” or “polyclonal.” In molecular epidemiology, the term “clone” is usually used to describe a group of independently isolated microorganisms that share so many phenotypic and genotypic characteristics that the most likely explanation is that they have a common origin (Struelens, 1996; van Belkum *et al.*, 2007). When isolates are characterized by WGS, a clone corresponds to a branch on a phylogenetic tree. In this context, a clone corresponds to the related concept “clade.” The common ancestor may be anywhere on the phylogenetic tree of life, and when studying populations one can define any number of subclones within clones. Therefore, in common usage the terms “clone” and “clade” are employed to define isolates having a *recent* common ancestor, with “recent” meaning bounded by genetic distance and/or epidemiologic parameters. Although there are no absolute boundaries of genetic distance in the common-use clone definition, the distance is informed by a range of ecological considerations, and is initially usually defined using organism-specific rule-of-thumb genetic distance cutoff values (e.g., alleles or SNPs) derived from outbreak surveillance data, as described earlier. These boundaries may widen or shrink as new epidemiological information becomes available to inform the “likeliness” of a common origin.

The ecology of enteric outbreaks can be complex, and interpretation of WGS for this purpose requires understanding of underlying assumptions and limitations, the use of graphical representations such as phylogenetic trees and similarity matrices, implications of analysis method and quality metrics, and approaches to detecting and triaging clusters.

## Disease and Outbreak Ecology

WGS is a powerful tool to help resolve epidemiological relationships, but must be interpreted in the context of the underlying disease and outbreak ecology. Enteric disease agents and resulting outbreaks vary widely in terms of their host range, reservoir, prevalence, mutation rates, environmental stability, epidemic potential, consumer behaviors, infective dose, complexity of the associated food chain, and transmission mechanisms. Outbreaks are classified by how the disease spreads through a population, and even seemingly simple outbreaks can be complicated when viewed at a molecular level. A “point-source” outbreak is a relatively straightforward transmission pathway characterized by a single point of introduction over a short period of time with most cases occurring within the same incubation period (Centers for Disease Control Prevention, 2006). A simple example is a group of restaurant patrons who become infected after consuming food contaminated on a single day by an ill restaurant food handler shedding a genetically homogeneous strain. It should be noted that the term “point source” refers to the transmission mechanism but not the agent, and does not necessarily imply transmission of a homogeneous or even a single agent. However, in this scenario, an infected food handler often serves as an evolutionary “bottleneck” resulting in low isolate diversity (Grad *et al.*, 2012), not unlike the process used in microbiology laboratories to obtain pure cultures by picking and passing individual colonies. The same type of genetic “bottleneck” may occur in other ecological niches.

Once established in each of the symptomatic or asymptomatic human hosts, the pathogen evolves to a greater or lesser degree along multiple separate evolutionary pathways. Replication occurs throughout the incubation period and course of illness, and the genome may be characterized at the point in the pathway where culture is performed. At the sequence level, the pathogens transmitted by the food handler and isolates detected in each of the patrons may differ from each other and from the initially transmitted pathogens, the degree of which is dependent on organism mutation rates and other factors. Therefore, the pathogen transmitted to the patrons may not be the same as the pathogen cultured from the food handler, as the transmission and detection steps may be separated by many rounds of replication. However, we would expect all of the isolates from this outbreak to be less different from each other than isolates that developed along an entirely different pathway, say, among cases in an unrelated outbreak.

Real-world foodborne and enteric disease outbreaks are often much more complex than the simple scenario described above. One or more agents or vehicles may be involved, and one, multiple, or continuous transmission events may occur that range from highly clonal, such as the restaurant scenario described above, to polyclonal events such as mass contamination of fruit due to inadequate disinfection of production equipment (McCollum *et al.*, 2013), to outbreaks involving *multiple species* or even multiple agents from different microbial kingdoms such as outbreaks due to fecal contamination of ground water (Gallay *et al.*, 2006). Zoonotic disease outbreaks, such as those associated with direct animal contact, may exhibit higher genetic diversity than isolates from a typical point-source outbreak (Basler *et al.*, 2016; Bosch *et al.*, 2016).

Adding to potential outbreak complexity, the chain of transmission may have links that are known, unknown, and unknowable. For example, Shiga toxin–producing *Escherichia coli* (STEC) can be passed from a primary reservoir such as cattle through food vehicles to people, from people to intermediary food vehicles, and a wide variety of other transmission mechanisms such as person to person, person to animal, animal- to person, and animal to plant (Medus *et al.*, 2006). Vehicles may migrate or mix, such as cattle carrying STEC that are transported, sold, and resold before slaughter, or produce lots that are intermingled before retail.

Different enteric disease agents have different host ranges, evolve at different rates, and survive and replicate variably in different environments, which in turn impacts transmission mechanisms and sequencing interpretation. The generation time of *E. coli* in culture can be as short as 15 min, which means that *E. coli* in a temperature-abused food theoretically may go through 96 rounds of replication per day. As a result, isolate diversity tends to increase over time. However, the opposite can also occur as bacteria may persist in a genetically quiescent state in biofilms or other sequestered environments. An extreme example of apparent bacterial quiescence is the 1998 and 2008 outbreaks of *Salmonella* Agona associated with dry cereal produced in a single processing plant where the PFGE type did not change in the intervening 10 years, potentially due to desiccation in a protected site within the production environment (Russo *et al.*, 2013). WGS is a powerful tool for teasing apart these complex relationships, but interpretation must always be made in the context of underlying ecology.

## Primary Assumptions and Their Limitations

WGS is a tool used to *infer* association or lack of association between illness-causing microorganisms found in people, specific foods, or food environments. However, association should not be confused with causality, and relationships inferred by characterizing pathogens are considered by themselves “hypotheses” not “proof.” WGS greatly increases the likelihood that the hypotheses are correct compared with earlier methods, but additional lines of evidence are always required to establish cause and effect. To understand this uncertainty, it is necessary to closely examine the implicit assumptions.

A central assumption underlying the use of WGS (or any subtyping method) for outbreak detection and investigation is that cases infected by pathogens that are phylogenetically close are *more likely* to have a recent common ancestor and have shared a common exposure (such as the same contaminated food or food supplier) than cases infected by strains that are more distantly related. The same assumption is used to link findings in people to those in foods, animals, or the environment. In the simple scenario above involving an ill food handler, we would expect the isolates from patrons, the food handler, and any positive isolates from implicated food vehicles to be indistinguishable or closely related (e.g., zero to five allele differences in a cgMLST analysis). Allele differences greater than zero are not unexpected among cases that are truly related, as the pathogens in each of the cases replicate and independently evolve to some degree. In addition, small sequencing or analysis errors may produce minor allele differences.

**FIG. 1.**
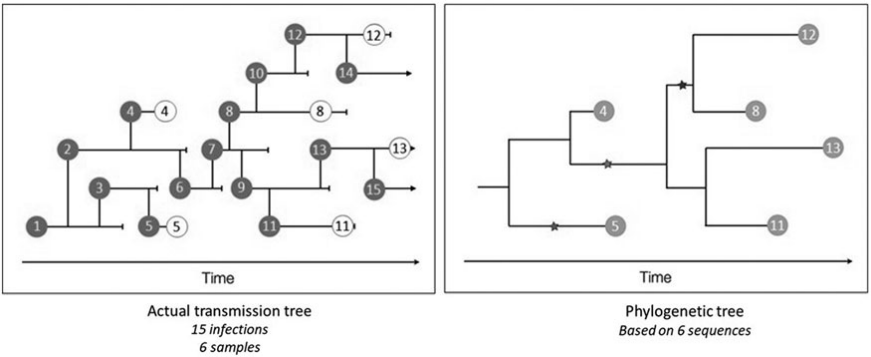
Transmission networks (left) include both sampled (dark gray circle) and unsampled (clear circle) events leading to a phylogenetic tree (right) based on only samples (light gray circle). (Courtesy of Trevor Bedford).

Close phylogenetic relatedness predicts *some* relationship between the people, or between people and foods, as the likelihood of random identity or convergent evolution among millions of base pairs of genetic code would be exceedingly low. However, the inference is not perfect because microbial phylogeny (the genetic relationship between strains) is not the same as epidemiological association (linkage in the chain of transmission). The relationship between phylogeny and transmission networks can be complex, and has been described elsewhere (Klinkenberg *et al.*, 2017). For purposes of this discussion, the important difference is that phylogenetic relatedness does not account for unrecognized intermediary steps in the chain of transmission. For example, a food handler harboring an outbreak strain may not have been directly responsible for the outbreak, but could have become ill from a contaminated and improperly stored ingredient in the restaurant, which was later consumed by patrons (Fig. 1). To determine cause and effect, other epidemiological information is necessary, such as information that patrons ate meals prepared by the food handler, became ill after an appropriate incubation period, and that data from other cases were not in conflict with the hypothesis.

The imperfect relationship between phylogeny and transmission due to unrecognized intermediates also limits the investigator's ability to predict the *direction* of transmission from phylogenetic information alone. In the simple scenario above, we would expect all isolates to have a common ancestor, but the isolate from the food handler may or may not be represented as the common ancestor even though he/she is a direct source of the patrons' illnesses (through food vehicles). This is because the pathogen involved in the transmission event may not be the same as the isolate detected in the food handler or the isolates detected in the patrons. In addition to potential pathogen genetic heterogeneity within each host, many rounds of replication may separate the transmission and detection events.

**Take Home Messages:**

- WGS relatedness can provide a hypothesis for association, but “proof” always requires some level of additional information.
- Highly related isolates are likely to have a recent common ancestor, but the direction of transmission cannot be assumed.

In epidemiological terms, WGS is primarily used to refine the case definition, which is the set of criteria used to specify which individuals are included in a study or outbreak investigation. This follows from the primary assumption that phylogenetic relatedness is proportional to likelihood of association. Case definitions are important for detecting and solving outbreaks because they are used to separate outbreak signals from background noise. The signal-to-noise ratio problem is most important when outbreak agents and outbreak vehicles are common (Besser, 2013). For example, egg-associated outbreaks due to *Salmonella enterica* serovar Enteritidis are difficult to solve in the United States without subtyping because both *Salmonella* Enteritidis infection and egg consumption are common. Any misclassified cases included in the study (e.g., ill people in an outbreak investigation who are not truly part of the outbreak) dilute measures of association, such as an odds ratio in a case–control study. However, if the agent is uncommon, the vehicle is uncommon, or there are a very large number of cases, subtyping may not be absolutely necessary. For example, the 2011 outbreak of listeriosis associated with cantaloupe involved multiple serotype and PFGE combinations, but listeriosis is a rare disease and the case count was high, allowing identification of the exposure before molecular analysis (Laksanalamai *et al.*, 2012; McCollum *et al.*, 2013). By grouping cases that are most likely to share an epidemiological association and excluding cases less likely to share an association, the strength of outbreak signals increases, and the number of cases needed to solve outbreaks is reduced. This allows outbreaks to be solved earlier when the opportunity for prevention is the highest.

**Take Home Message:**

- WGS improves our ability to detect and solve outbreaks, but it is not always necessary to “prove” an association.

A second cardinal assumption is that outbreaks are primarily clonal. While some outbreaks are clearly clonal, as described earlier this is not always the circumstance, which means that the existence of genetic distance cutoff values for inclusion or exclusion of cases from outbreaks that would be applicable for every setting is a biological impossibility. However, for practical detection of widespread outbreaks we assume that at least some subset of cases appear monoclonal, and these cases are used to focus the ensuing epidemiological investigation.

Once a cluster is detected and supporting epidemiological and trace-back information is gathered, it may be justified to expand the WGS-based case definition (stringency lowered) to determine if additional cases can be found that may be outbreak associated, which increases the study sensitivity. Increasing sensitivity may be important for solving outbreaks when they are small, for linking outbreak data to other types of information, such as product testing and trace-back data, and for determining the scope of an outbreak (Reingold, 1998). Expanding the case definition can be accomplished with genomic data by evaluating cases in different tree nodes moving from the “leaves” toward the “trunk,” or by using different digits in strain nomenclature such as SNP addresses used by Public Health England (Inns *et al.*, 2017) or “allele codes” used by PulseNet USA (Nadon *et al.*, 2017). Expansion of the case definition can also be triggered by other types of laboratory or epidemiology data, such as culture findings from implicated food or epidemiologically linked cases.

Although clustering by whole-genome phylogeny has become the standard in foodborne disease outbreak detection, WGS also provides an opportunity to detect clusters related only by other factors such as antibiotic resistance or plasmid content. For example, in the 2016–2018 outbreak of multidrug-resistant *Campylobacter jejuni* associated with puppy exposure, a common thread was antibiotic prescribing practices, which presented as a common resistance profile in genetically diverse isolates (e.g., multiple clades with large allele differences) (Montgomery *et al.*, 2018). Although nature often defies our desire for simplicity, the flexibility, resolution, and evolutionary model of WGS uniquely provides tools needed to unravel nature's complexities for public health action.

**Take Home Messages:**

- Genetic distance cutoff values are useful tools for WGS cluster detection, but cannot be used by themselves to definitively exclude cases.
- WGS makes it possible to adjust the specificity and sensitivity of the case definition to answer different questions in the course of outbreak detection and investigation.

## Understanding and Using Phylogenetic Trees

Tree diagrams are used to visualize the relationship between genomes, and commonly serve as primary outbreak and investigation tools. The process of tree building starts with cluster analyses including distance-based hierarchical methods (e.g., single linkage, UPGMA), which are used for character (alphanumeric) data generated for gene level analysis such as cgMLST, and nonhierarchical methods (e.g., maximum likelihood), which are employed with sequence data (e.g., A,T,C, and G's) used for SNP analyses. Details of clustering methods are presented elsewhere (Baldauf, 2003). Tree diagrams may be rooted, which means that the most recent common ancestor of all the isolates in the analysis group is inferred, and represented as the “ancestral node.” Unrooted trees show relatedness between strains without inferring ancestry. Rooted trees are best for visualizing the relationships between isolates, and are the most commonly used representation in foodborne disease surveillance. Unrooted trees are preferred for visualizing broad trends such as clonal expansion in time or space.

**FIG. 2.**
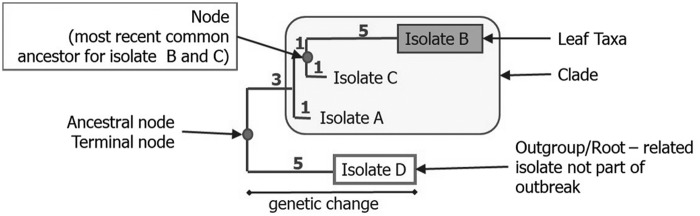
Anatomy of a phylogenetic tree: horizontal lines and numbers represent relative genetic distance.

**FIG. 3.**
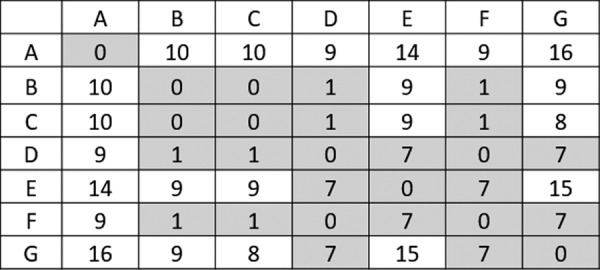
An allele matrix, pairwise comparison of isolates. Shading is based on a cutoff of <7 allele differences.

In rooted tree diagrams, isolates are most commonly depicted as the “leaves” at the end of horizontal “branches” whose length is proportional to genetic distance to a “node” or theoretically most common ancestor (Fig. 2). In this depiction, the vertical lines represent the relationship between “branches,” but their lengths are arbitrary. Numbers shown on vertical lines may represent median and range of allele differences (e.g., cgMLST analysis) or SNP differences (e.g., hqSNP analysis). Trees generated from SNP analyses may include “bootstrap” values, which predict the likelihood that the grouping is correct as represented. The “tree” does not predict which isolate represents a common ancestor to another isolate due to inherent uncertainty, as described earlier. The limitations of two-dimensional representations make it possible for two isolates to be more “related” to each other than a third isolate due to their sharing a recent common ancestor, but more “similar” to the third isolate, which has less genetic differences. What this means for interpretation is that the broad trends may be true (e.g., which genomes are generally related), but other types of information, such as epidemiological data, are needed to further clarify specific relationships. SNP or allele matrices (Fig. 3) present pairwise differences between isolates, but are not used to visualize the overall population structure.

## How Does WGS Differ from Earlier Subtyping Methods, and Does “Whole-Genome Sequencing” Involve the Whole Genome?

Earlier methods such as PFGE and multilocus variable number tandem repeat analysis (MLVA) share with WGS the basic assumptions described above. WGS differs from the earlier methods primarily in the strength of phylogenies built on the molecular data and strain resolution. Phylogenies are built upon mathematical models of evolutionary relationships, and the strength of the evolutionary model is not equal for all subtyping methods. Although criteria were developed in the 1990s for assessing the relatedness of isolates using PFGE band differences (Tenover *et al.*, 1995), the method was based on indirect inference of the underlying sequence information, which introduces considerable uncertainty. For this reason, the use of the relatedness measures other than “indistinguishable” for PFGE has been discouraged in PulseNet (Barrett *et al.*, 2006). Without the option to include “closely related” strains in the case definition, a larger number of total cases are needed with PFGE to detect outbreak signals that can be acted upon. WGS methods use sequence data directly to construct phylogenies, greatly reducing uncertainty and allowing inclusion of closely related strains in the case definition. This flexibility makes it possible to detect and solve very small outbreaks with two to three cases. The greater resolution of WGS also reduces the number of cases needed to detect and solve outbreaks. Greater resolution makes it possible to exclude cases less likely to share an epidemiological association, which in turn increases the strength of association measures.

It should be noted that most (but not 100%) of the DNA present in a sample is sequenced, but not all regions can be assembled with current technology, and different types of WGS analyses utilize more or less of the available sequence information. Loci evaluated in a cgMLST analysis are generally limited to predefined coding regions that are common (within some tolerance level) to all members of the taxonomic group of interest, and generally do not include mobile elements and other less stable loci. wgMLST uses a greater proportion of the genome, which can be helpful in answering certain epidemiological questions, but can also introduce uncertainty due to overclassification and instability. SNP analyses are not limited to predefined or coding loci, but are limited to regions that each members of the query group share with the reference genome used for SNP calling. If the required reference strain is not close to the query isolates the number of shared loci will be small. In addition, only single-nucleotide polymorphisms are evaluated, mobile elements and other rapidly evolving regions are generally excluded from SNP analyses, and in some analyses only loci shared among all members of the query group are considered. For both MLST and SNP analyses, only assembled regions are assessed. With WGS, multiple types of sequence analyses may be performed as investigators are not limited to one particular analysis or one level of resolution.

## How Much Resolution Is Needed? Is More Better?

As one increases the proportion of the genetic content used in a WGS analysis, an increasing amount of variation is captured. If too little or the wrong information is captured by the analysis, outbreak signals are obscured due to inclusion of unrelated cases in the study that create statistical noise (e.g., unexplained variability). Too much resolution results in overclassification, a different sort of noise, wherein every case potentially appears different from every other case. To make sense of this type of information, some sort of regrouping of the data is necessary. For this reason, rule-of-thumb relatedness cutoff values are used. For this process to be effective, it is necessary for the genetic elements used in the analysis fit into the phylogenetic model. For example, although mobile elements represent a potentially useful source of variation in bacteria they are generally excluded from analyses because mutations due to mobile elements occur at a different rate than chromosomal elements, increase uncertainty in the analysis, and using them in the phylogeny may distort the represented genetic relationships. However, mobile elements are one of the primary markers in PFGE and contribute to its sensitivity (Jordan and Dalmasso, 2015).

The optimal amount and type of variation required depend on the epidemiological question being asked. For example, it has been proposed that WGS may be useful for food source attribution, but only by limiting the amount and type of data used to avoid a deleterious signal to noise ratio (de Knegt *et al.*, 2016). Groups have been using machine learning methods to identify specific genomic loci for use in this type of analysis (Zhang *et al.*, 2019), excluding potentially confounding noise. Therefore, “more” is not necessarily “better,” and the right amount of resolution depends on the question being asked.

In PulseNet International, gene-by-gene approaches such as cgMLST have been proposed as the strain-typing information most likely to be useful for identifying dispersed outbreaks (Nadon *et al.*, 2017). In situations where specific epidemiological questions need to be addressed, additional analyses may be used. For example, if a subcluster of cases nearly identical by cgMLST appear to differ from other cases in some way, such as having different demographic, antibiotic usage, exposure, or geographic characteristics, systematic differences can be explored by adding other types of analyses, such as wgMLST, SNP analysis, or plasmid analysis.

Finally in some situations, phylogeny has been used as a “molecular clock.” For example, sequence data were used to estimate elapsed time from exposure to sample collection in a patient infected with polio from a vaccine-derived strain (Alexander *et al.*, 2009). While such analyses can theoretically be conducted with foodborne agents such as *Salmonella* or STEC, investigators should take into account varying growth and mutation rates in known or unknown environments and transmission steps, which could confound attempts at interpretation.

**Take Home Message:**

- The optimal amount of resolution in WGS analysis depends on the question being asked, and more is not necessarily better.

## How Does “DNA Fingerprinting” of Pathogens by WGS Differ from “DNA Fingerprinting” Methods Used for Humans?

WGS greatly increases confidence in strain associations when compared with earlier methods, but can confidence reach levels seen in “DNA fingerprinting” used for human forensics, and can confidence be similarly quantified? Both activities are used to assess similarity or distance between genomes, and both require other lines of evidence to “prove” an association. However, in human populations the denominator (number of humans) and allele frequencies can be accurately estimated and are fairly stable, making it possible to calculate the probability that two samples are from the same person (or an identical twin) to a high degree of certainty using an MLVA-like analysis. In microbial populations, it is possible to calculate the likelihood of genetic association within a highly defined population, such as specific isolates in a phylogenetic analysis (see earlier “bootstrap value” discussion), but it is not possible to assess allele frequencies in the broader microbial populations due to constant genetic change, complex ecology, and an unknown denominator. It is therefore not possible to calculate an exact probability of the relationship of two isolates from their allelic profile. In addition, human DNA does not replicate outside the human body, and most genetic changes occur during sexual reproduction, with a generation time of ∼25 years, while microbial life may replicate in multiple hosts or environments, have complex transmission pathways with known and unknown links, and generation times that are extremely short when measured against our own.

While the chance of error of a WGS match cannot be independently calculated, the *p*-value of analytical studies represents the probability that the association (e.g., between a group of illnesses and a particular food) can be explained by chance alone. For example, a *p*-value of 0.001 is equivalent to saying that there is a 1 in 1000 chance of statistical error. As in any epidemiological investigation, confounding and other types of errors must also be considered. In conclusion, while DNA “fingerprinting” methods are used for both human forensics and foodborne disease surveillance and outbreak detection, interpretation for human forensics is more straightforward and quantifiable due to reduced levels of inference, a simpler ecology, and defined allelic denominator.

## Interpretation of Individual Findings Within a WGS-Defined Cluster (Including Historical Findings)

Due to the high specificity and evolutionary models used, there is less opportunity for misclassification of cases using WGS analysis than earlier methods. Therefore, “matches” between isolates strongly suggest *some* relationship, whether separated in time. For example, WGS is making it possible to recognize persistence problems due to contaminated food production or preparation environments (Elson *et al.*, 2019).

Although conclusions can be reached about groups of cases with high certainty in outbreaks, the same cannot be automatically assumed about any individual finding within the group. In statistics, this is known as the ecological fallacy. What this means in practical terms is that simply being part of a WGS cluster (e.g., a cluster of human cases with or without food/environmental findings) strongly suggests a common link but does not by itself prove an association with the rest of the group. This is due, as described earlier, to potential unrecognized intermediates that could confound a hypothesis of direct connection. Therefore, some level of additional information is needed. How much additional information depends on multiple factors, such as the commonness of the agent or the suspected vehicle (Besser, 2013). For example, for a WGS cluster involving a relatively rare exposure and/or agent it may be sufficient to know that a case consumed or could have consumed the implicated product, but the same may not be true for a very common agent or common exposure.

**Take Home Messages:**

- Although common exposures in outbreaks can be identified to a high degree of certainty, association cannot be automatically assumed for each individual case-patient based on microbiology data alone …. some additional evidence is required.

## Analysis Quality and Interpretation

The quality of WGS analyses can impact interpretation by increasing false association or nonassociation of isolates. Including poor sequences, for example, with borderline-to-low coverage, poor-quality scores, or shorter than expected average read lengths, in the SNP analysis can result in fewer positions being considered for pairwise comparisons, and hence reduced resolution and increased likelihood of false association. Contamination, particularly same-species contamination (e.g., *Salmonella* with *Salmonella*), can cause artificial cgMLST allele profiles, and result in erroneous placement of the sequence in the phylogeny and false association and/or nonassociation. It is therefore important to evaluate quality metrics, not just for the raw sequences entering the analyses but also for the analyses themselves. This is especially important when sequences to be included in the analysis are generated by multiple participants in a network of laboratories.

Postanalysis metrics such as percentage of reads mapped back to the reference, percentage of bases masked, and the number of alleles detected can give clues about the reliability of the analysis. For SNP analysis, it is also imperative to keep in mind that incorrect choice of the reference sequence (i.e., not closely enough related to the study population) will result in postanalysis quality metrics that are similar (poor mapping back to the reference, high masking percentage) to using sequences of suboptimal quality in the analysis.

As a matter of principle, only sequences that pass minimum-quality standards should be included in WGS analyses. However, in urgent outbreak settings it may be necessary to use sequences of suboptimal quality for preliminary analyses until repeat sequencing can be performed. In that circumstance, the limitations and potential interpretation pitfalls should be clearly communicated.

## Practical Cluster Detection and Triage

Foodborne disease clusters are generally recognized by a common exposure such as an event, or by the use of pathogen-specific laboratory data such as WGS typing results that are grouped in time or space. Given the complex ecology of foodborne disease agents, clusters may be defined in an almost infinite variety of ways. Practical cluster detection involves the use of specific criteria to focus attention on signals most likely to be actionable. These criteria typically include (1) a minimum threshold for number of cases, (2) maximum allowed SNP or allele differences between isolates in a presumed cluster based on a particular agent and typing method, and (3) a specific time interval. For example, parameters established by Moura *et al.* for listeriosis clusters in France include (1) two or more cases, (2) up to seven allele differences based on cgMLST, and (3) a 2-year time period. The allelic difference threshold was empirically determined by comparing the genetic heterogeneity between epidemiologically related and unrelated isolates (Moura *et al.*, 2016, 2017).

The minimum threshold for number of cases to investigate also decreases if matching food or environmental isolates are available. Due to the high specificity of WGS, the matching food source often proves to be the source of the outbreak. In general, the decision on where to draw thresholds depends on the specific agent (e.g., species, serotype) and its genetic diversity, ecology, and prevalence. Severity of illness may also contribute to cluster definitions. For example, two cases of botulism occurring in a similar time frame or location may be considered as a cluster without regard to genetic distance due to large potential public health consequences, whereas those criteria would probably not be used if the agent were *Salmonella enterica* serotype undetermined. CDC established working cgMLST cluster definitions in PulseNet for *L. monocytogenes*, *Salmonella*, and STEC using foodborne and zoonotic outbreaks initially identified through PFGE and other mechanisms in parallel with WGS, empirically widening or narrowing cgMLST cluster definitions as needed to capture the maximum number of events (CDC, unpublished data). For example, a working definition for *Salmonella* includes ≥3 cases in a 60-day window with 0–10 allele differences, where ∼2 cases are related by at most 5 differences. Genetic diversity varies by *Salmonella* serovar, which helps inform triage decisions and ultimately may drive establishment of serovar-specific working thresholds. The allelic threshold was broadened slightly to capture zoonotic disease outbreak clusters, which as described earlier typically have greater allelic diversity than foodborne outbreaks.

Raising distance thresholds not only increases detection sensitivity since more true outbreaks are captured, but also decreases specificity due to the possible inclusion of misclassified cases. Such cases dilute measures of association, thereby decreasing the likelihood of a successful investigation. At CDC historical sequences within 15 alleles of the cluster are added to the cluster report to enhance hypothesis generation. Due to the likelihood of persistent low-level food contamination in food production environments and the high specificity of WGS, the use of time as a defining criterion may be re-evaluated. Clusters are also identified by real-time examination of phylogenetic trees for groups of case isolates that stand out from background cases. The process is currently visual, but may be amenable to statistical tools such as fixation indices, which measure variation within and between populations (Holsinger and Weir, 2009).

In the course of investigations, additional subtyping methods such as wgMLST or hqSNP analysis may be performed to explore alternative hypotheses. The use of WGS as a primary surveillance tool is decreasing the size and increasing the number of clusters that can be effectively investigated, thus escalating the need for triage. Cluster triage is currently based on public health threat and recognition of patterns in time, space, or case attributes that are suggestive of previous outbreaks or patterns of disease that are different than what is expected.

## Future Directions

WGS involves increasing the amount of data available to detect and monitor trends by orders of magnitude, which will likely lead to new prevention opportunities. It is expanding the very concept of a “cluster” and an “outbreak,” and will require us to reconsider traditional hypothesis generation methods. Advanced analytical tools such as anomaly detection and machine learning are needed to integrate genomic and epidemiology data streams to aid in interpretation and to exploit the full potential of WGS.

## Disclaimer

The findings and conclusions in this presentation are those of the author and do not necessarily represent the views of the Centers for Disease Control and Prevention.
